# U-73122 reduces the cell growth in cultured MG-63 ostesarcoma cell line involving Phosphoinositide-specific Phospholipases C

**DOI:** 10.1186/s40064-016-1768-6

**Published:** 2016-02-24

**Authors:** Vincenza Rita Lo Vasco, Martina Leopizzi, Valeria Di Maio, Carlo Della Rocca

**Affiliations:** Sensory Organs Department, Policlinico Umberto I, Faculty of Medicine and Dentistry, Sapienza University of Rome, viale dell’Università, 33, 00157 Rome, Italy; Medico-Surgical Sciences and Biotechnology Department, Polo Pontino- Sapienza University of Rome, 04100 Latina, Italy

**Keywords:** Oncology, Osteosarcoma, MG-63, Phosphoinositide, Phospholipase C, U-73122, U-73343, DMSO

## Abstract

The definition of the number and nature of the signal transduction pathways involved in the pathogenesis and the identification of the molecules promoting metastasis spread might improve the knowledge of the natural history of osteosarcoma, also allowing refine the prognosis and opening the way to novel therapeutic strategies. Phosphatydil inositol (4,5) bisphosphate (PIP2), belonging to the Phosphoinositide (PI) signal transduction pathway, was related to the regulation of ezrin, an ezrin–radixin–moesin protein involved in metastatic osteosarcoma spread. The levels of PIP2 are regulated by means of the PI-specific Phospholipase C (PLC) enzymes. Recent literature data suggested that in osteosarcoma the panel of expression of PLC isoforms varies in a complex and unclear manner and is related to ezrin, probably networking with Ras GTPases, such as RhoA and Rac1. We analyzed the expression and the subcellular localization of PLC enzymes in cultured human osteosarcoma MG-63 cells, commonly used as an experimental model for human osteoblasts, using U-73122 PLC inhibitor, U-73343 inactive analogue, and by silencing ezrin. The treatment with U-73122 significantly reduces the number of MG-63 viable cells and contemporarily modifies the expression and the subcellular localization of selected PLC isoforms. U-73122 reduces the cell growth in cultured MG-63 ostesarcoma cell line involving PI-specific Phospholipases C.

## Background

Osteosarcoma comprises less than 1 % of cancers in the United States, occurring in less than 1000 patients per year. It is the most common primary bone tumour in childhood and adolescence (Mirabello et al. 2009a, b). The presence of metastasis confers worse prognosis for osteosarcoma affected patients (Meyers et al. 2005). Metastasizing tumours partially respond to current therapies and represent the primary cause of cancer related mortality (Meyers et al. 2005).

Osteosarcoma includes several pathological entities, comprising of different clinical, radiological, and histological features (Mirabello et al. 2009a, b; Gatta et al. 2005). Osteosarcoma arises from a mesenchymal cell that owns or can acquire the ability to produce osteoid (Gorlick et al. 2003; Marina et al. 2010). The complex pathogenesis of osteosarcoma includes numerous factors (Gorlick et al. 2003), both genetic abnormalities and environmental exposures, in experimental models as well as in humans (Gorlick 2009). Many efforts to identify a unifying recurrent event were made, bringing multiple genetic risk factors together with epidemiologic and association data. However, despite the considerable improvement in the knowledge, the pathogenesis of osteosarcoma is largely unknown.

The definition of the number and nature of the signal transduction pathways involved in the pathogenesis and the identification of the molecules promoting metastasis spread might improve the knowledge of the natural history of osteosarcoma, allowing refine the prognosis and opening the way to novel therapeutic strategies. Recently, great interest arose about ezrin a protein belonging to the ezrin–radixin–moesin (ERM) family, and related signal transduction pathways (Khanna et al. 2004; Ferrari et al. 2008; Hunter 2004, Dard et al. 2004; Zhu et al. 2007; Yang et al. 2012; Zhao et al. 2011; Tan and Yang 2010). Ezrin protein is codified by *Vil2* gene (OMIM *123900). The Protein 4.1, ezrin, radixin, moesin (FERM) domain (Chishti et al. 1998) of ezrin is involved in the recognition of Phosphatydil inositol (4,5) bisphosphate (PIP2), a crucial molecule belonging to the Phosphoinositide (PI) signal transduction pathway (Gautreau et al^1999^; Martin 2003; Pujuguet et al. 2003; Zhao et al. 2004; Hao et al. 1997; Fievet et al. 2004, 2007). The actin binding activity of Ezrin (Defacque et al. 2000, 2002) largely depends on the membrane PIP2 levels (Hao et al. 2009). ERM proteins simultaneously bind actin and, by means of their N-terminal domains, PIP2 located at the membrane (Niggli and Rossy 2008; Gilmore and Burridge 1996; Isenberg and Niggli 1998; Nakamura et al. 1999; Eberle et al. 1990; Dobos et al. 1992; Apgar 1995; Hartwig et al. 1995; Gachet et al. 1997; Gratacap et al. 1998). Beside phosphorylation, activation of ERM proteins, was suggested to occur after interaction with PIP2, which induces the conformation to open (Gilmore and Burridge 1996). Both PIP2 binding and phosphorylation are thought to allow the stabilization of ERM proteins or a more efficient binding to their own receptors (Hirao et al. 1996; Heiska et al. 1998; Legg and Isacke 1998; Nakamura et al. 1999). Increasing evidences indicated that ezrin is involved in osteosarcoma progression and metastasis and that the levels of PIP2 play a critical role for its activation.

PIP2, a phosphorylated derivative of phosphatydil inositol mainly located in the inner half of the plasma membrane lipid bilayer, is critical for many cellular activities, such as endo- and exocytosis, ion channel activity and cell motility. The levels of PIP2 are regulated by means of PI-specific Phospholipase C (PLC) family of enzymes (Berridge and Dupont 1994; Divecha and Irvine 1995; Hisatsune et al. 2005; Rhee 2001; Bunney and Katan 2011; Fukami et al. 2010).

Activated PLC cleaves PIP2 into inositol trisphosphate (IP3) and diacylglycerol (DAG), both crucial molecules in signal transduction (Rhee et al. 1991). IP3 induces calcium release. DAG can be further cleaved to release arachidonic acid (Tang et al. 2005) or can activate serine/threonine calcium-dependent protein kinase C enzymes (PKC), also influenced by the IP3-induced calcium increase.

The mammalian PLC family comprises a related group of complex, modular, multi-domain enzymes which cover a broad spectrum of regulatory interactions, including direct binding to G protein subunits, small GTPases from Rho and Ras families, receptor and non-receptor tyrosine kinases and lipid components of cellular membranes (Rhee et al. 1991). PLC enzymes are thirteen isoforms classified on the basis of amino acid sequence, domain structure and mechanism of recruitment into six subfamilies: β(1–4), γ(1–2), δ(1, 3, 4), ε(1), ζ(1), and η(1–2) (Suh et al. 2008).

The activity of PLC is required for chemokine mediated dissociation of ERM proteins from the membrane (Brown et al. 2011). Previous studies had placed selected PLC enzymes at the convergence point for the broad range of signalling pathways that promote Rho and Ras GTPase mediated signalling (Hao et al. 2009; Lo Vasco et al. 2015), which also contributes to the regulation of ezrin metabolism. In our previous reports we suggested that the RasGTPases network ezrin involving the PLC enzymes (Lo Vasco et al. 2015).

In our previous reports, we identified the panel of expression of PLC enzymes (Lo Vasco et al. 2013) and analyzed the effect of ezrin silencing or *PLCE* isoform silencing upon selected osteosarcoma cell lines (Lo Vasco et al. 2014a, b).

In the present experiments, we analyzed the PLC signal transduction system in cultured human osteosarcoma MG-63 cells. MG-63 cell line is commonly used as an experimental model for human osteoblasts, presenting with low levels of alkaline phosphatase activity, and PTH unresponsive adenylate cyclase (Fukayama and Tashjian Jr. 1990).

We treated MG-63 cells with U-73122 (1-[6-[[17b-3-methoxyestra-1,3,5(10)-trien-17-yl]amino]exyl]-1*H*-pyrrole-2,5-dione), a widely used PLC enzyme inhibitor that probably also acts upon the gene regulation (Lo Vasco et al. 2010a, 2013 os1). U-73343 [1-(6-((17-beta-3-methoxyestra-1,3,5(10)-trien-17-yl)amino)hexyl)-2,5-pyrrolidinedione], a structural analogue of U73122, with negligible activity as a PLC inhibitor, was used as a control compound (Heemskerk et al. 1997). We silenced *Vil2* using siRNA methodology (Lo Vasco et al. 2014a). We evaluated all those treatments upon the MG-63 viability, in order to investigate the role of PLC inhibition upon cell growth and survival. As U-73122 is not water soluble and it used to be dissolved in dimethyl sulfoxide (DMSO), we evaluated the effects of DMSO alone upon cultures. We also evaluated the morphological changes occurring in MG-63 after different treatments and investigated the localization and sub-cellular distribution of PLC isoforms within untreated and U-73122 treated MG-63 cell.

## Methods

### Cell culture

MG-63 human osteosarcoma cell line was obtained from the American Type Culture Collection (ATCC, Rockville, MD, USA). Cells were counted using a Neubauer haemocytometer and a phase contrast microscope. Cells were grown at 37 °C with 5 % of CO_2_ in Dulbecco’s minimum essential medium (Sigma) supplemented with 10 % fetal bovine serum (GIBCO), penicillin (100 μg/ml), streptomycin (100 U/ml) and sodium pyruvate. Cells were grown for 24 h, reaching a confluence of around 40–60 %, and until confluence (reached from 72 to 96 h). U-73122 (Sigma-Aldrich) was addicted solved in DMSO (Takenouchi et al. 2005; Lo Vasco et al. 2011).

The initial number of cells (time 0) was 250.000 for each experiment of growth curve assessment. The number of cells for molecular biology experiments was 1 × 10^6^ cells/each experiment. Each experiment was repeated at least three times.

Cells were grown under different conditions. Cells were grown respectively 24 h: (1st) without treatment, with addiction to the culture medium of respectively (2nd) DMSO (3th) 10 μΜ of U-73122, (4th) 10 μM U-73343 (Sigma-Aldrich), (5th) 1 μM U-73122. Untreated cells were also grown (6th) until the confluence was reached (range 72–96 h). Analyses of cultures was performed, both in treated and untreated cells, in the beginning of experiment (time 0), after 1, 3, 6, 24 h and at confluence for PCR experiments; after 18 and 24 h for morphology experiments. Experiments were independently repeated at least 3 times for each isoform.

### Cell survival Trypan blue test

Cells suspension was diluted 1:1 in 0.4 % Trypan blue staining (Sigma Aldrich, Dorset, UK) for survival quantification. Viable cells were counted using a Neubauer haemocytometer and a phase contrast microscope. The following equation was used to calculate the total number of viable cells in 1 ml suspension: number of total viable cells in 1 ml (TC) = $\bar{x}*2*10^{4}$ ($\bar{x}$ = average of the cell counts from the squares of the haemocytometer grid, 2 = dilution factor 1:1). The number of live cells was used to determine the growth rate and experiments were repeated three times.

### Cells transfection for ezrin silencing

MG63 cells were transiently transfected with ezrin silencing RNA using METAFECTENE SI+ (Biontex Laboratories GmbH, Munich, Germany). siRNA sequences targeting Ezrin and negative control siRNA, were designed and synthesized by Invitrogen (Life Technologies, Foster City, CA, USA). The siRNA was designed according to Ezrin complementary DNA (cDNA) sequence (EZR Gene ID: 7430). Briefly, 2.2 ml cell suspension were prepared in complete cell culture medium with a concentration of 1.5 × 10^5^ cells/ml. Cells were seeded, in 6-well plates, shortly before the addition of the lipoplex, according to the manufacturer’s instructions. Then cells were incubated under normal culture conditions (37 °C in CO2–containing atmosphere) until the lipoplex addition. Before transfection, 150 µl of 1× SI+ buffer were mixed with 72 µl of METAFECTENE^®^ SI+ and 540 pMol of RNA stock solution. The mixture was incubated for 15 min at room temperature and then added to the cells in 1 h from seeding. Cells were incubated 72 h. Functional siRNA was measured by reverse transcription–polymerase chain reaction (RT-PCR) and western blot analysis 24, 48 and 72 h after tranfection.

### RNA extraction

After all the above indicated treatment procedures and times, cells were detached and suspended using TRIzol reagent (Invitrogen Corporation, Carlsbad, CA). Total RNA was extracted with a SV Total RNA Isolation System (Promega, Madison, WI, USA) according to the manufacturer’s instructions. The concentration and purity of the obtained RNA was checked using a NanoDrop ND-1000 Spectrophotometer (Thermo Fisher Scientific, Inc. USA).

### RT-PCR

RNA was reverse-transcribed into cDNA using High-Capacity cDNA Reverse Transcription Kit (Life Technologies, Foster City, CA, USA) following manufacturer’s indications. The RNA mix was then amplified for 10 min at 25 °C, 120 min at 37 °C and 5 min at 85 °C in a Gene Amp^®^ PCR System 9700 (Applied Biosystems) thermocycler.

Glyceraldehyde 3 phosphate dehydrogenase (*GAPDH*) was used as positive control (Bio Basic Inc, Amherst, New York, USA). The primer pairs (Bio Basic Inc, Amherst, New York, USA) for each PLC isoform, *GAPDH* and *Vil2* gene are listed in Table 1. The specificity of the primers was verified by searching in the NCBI database for possible homology to cDNAs of unrelated proteins. RNA samples were also amplified by PCR without RT to exclude possible contamination.

**Table 1:** PCR primers

PI-PLC β1 (PLCB1; OMIM *607120)	Forward 5′-AGCTCTCAGAACAAGCCTCCAACA-3′ Reverse 5′-ATCATCGTCGTCGTCACTTTCCGT-3′
PI-PLC β2 (PLCB2; OMIM *604114)	Forward 5′-AAGGTGAAGGCCTATCTGAGCCAA-3′ Reverse 5′-CTTGGCAAACTTCCCAAAGCGAGT-3′
PI-PLC β3 (PLCB3; OMIM *600230)	Forward 5′-TATCTTCTTGGACCTGCTGACCGT-3′ Reverse 5′-TGTGCCCTCATCTGTAGTTGGCTT-3′
PI-PLC β4 (PLCB4; OMIM *600810)	Forward 5′-GCACAGCACACAAAGGAATGGTCA-3′ Reverse 5′-CGCATTTCCTTGCTTTCCCTGTCA-3′
PI-PLC γ1 (PLCG1; OMIM *172420)	Forward 5′-TCTACCTGGAGGACCCTGTGAA-3′ Reverse 5′-CCAGAAAGAGAG CGTGTAGTCG-3′
PI-PLC γ2 (PLCG2; OMIM *600220)	Forward 5′-AGTACATGCAGATGAATCACGC-3′ Reverse 5′-ACCTGAATCCTGATTTGACTGC-3′
PI-PLC δ1 (PLCD1; OMIM *602142)	Forward 5′-CTGAGCGTGTGGTTCCAGC-3′ Reverse 5′-CAGGCCCTCGGACTGGT-3′
PI-PLC δ3 (PLCD3; OMIM *608795)	Forward 5′-CCAGAACCACTCTCAGCATCCA-3′ Reverse 5′-GCCA TTGTTGAGCACGTAGTCAG-3′
PI-PLC δ4 (PLCD4; OMIM *605939)	Forward 5′-AGACACGTCCCAGTCTGGAACC- 3′ Reverse 5′-CTGCTTCCTCTTCCTCATATTC- 3′
PI-PLC ε (PLCE; OMIM *608414)	Forward 5′-GGGGCCACGGTCATCCAC-3′ Reverse 5′-GGGCCTTCATACCGTCCATCCTC-3′
PI-PLC η1 (PLCH1; OMIM *612835	Forward 5′-CTTTGGTTCGGTTCCTTGTGTGG-3′ Reverse 5′-GGATGCTTCTGTCAGTCCTTCC-3′
PI-PLC η2 (PLCH2; OMIM *612836)	Forward 5′-GAAACTGGCCTCCAAACACTGCCCGCCG-3′ Reverse 5′-GTCTTGTTGGAGATGCACGTGCCCCTTGC-3′
Glyceraldehyde 3 phosphate dehydrogenase (GAPDH)	Forward 5′-CGAGATCCCTCCAAAATCAA-3′ Reverse 5′-GTCTTCTGGGTGGCAGTGAT-3′

Standard analytical PCR reaction was performed with GoTaq Master Mix (Promega) following manufacturer’s instructions. Cycling conditions were performed with 95 °C initial denaturation step for 1 min was followed by 40 cycles consisting of 95 °C denaturation (30 s), annealing (30 s) at the appropriate temperature for each primer pair and 72 °C extension (1 min) in Gene Amp^®^ PCR System 9700 (Applied Biosystems) thermocycler. Amplified PCR products were analysed by 1.5 % TAE ethidium bromide-stained agarose gel electrophoresis (Agarose Gel Unit, Bio-Rad Laboratories S.r.l., Segrate, IT). A PC-assisted CCD camera (GelDoc 2000 System/Quantity One Software; Bio-Rad) was used for gel documentation and quantification. Optical densities were normalized to the mRNA content of *GAPDH*.

RNA samples were also amplified by PCR without RT. No band was observed, excluding DNA contamination during the procedure (data not shown). Experiments were independently repeated at least 3 times for each isoform.

### Real-time PCR

Gene expression of Ezrin, PLC ε, PLC γ2, PLC δ4 were determined by real-time PCR using the 7500 Real-Time PCR instrument from Applied Biosystems™. TaqMan^®^ primers and probes for each gene, as well as the GAPDH reference gene, were obtained from Applied Biosystems™.

Transfected MG63 cells and normal controls were harvested 24, 48 and 72 h after transfection. The messenger RNA (mRNA) expression of Ezrin, PLC ε, PLC γ2, PLC δ4 was determined by Real-Time PCR. Total RNA was extracted with a SV Total RNA Isolation System (Promega, Madison, WI, USA) according to the manufacturer’s instructions. We confirmed purity and quantity of RNA by NanoDrop ND-1000 Spectrophotometer (Thermo Fisher Scientific, Inc. USA). The RNA was reverse transcribed into cDNA with High Capacity cDNA Reverse Transcription Kit (Life Technologies, Foster City, CA, USA).

PCR products were detected using gene-specific primers and probes labeled with reporter day FAM which yielded a predicted amplicons of 82, 84, 61, 78, 64, 93 and 62 base pairs respectively; glyceraldehyde-3-phosphate dehydrogenase (*GAPDH*) was used as an internal standard, which yielded a predicted amplicon of 58 base pairs. Reaction mixtures for all gene expression assays contained: 5 μl TaqMan^®^ mastermix (2×; Applied Biosystems™), 0.5 μl gene of interest primer/probe mix and 1 μl PCR grade water. To each reaction, 3.5 μl of the diluted cDNA (35 ng) were added. All samples were assayed in triplicate. PCR reaction was carried out in triplicate on 96-well plate with 10 μl per well using 1× TaqMan Master Mix. After an incubation for 2 min at 50 °C and 10 min at 95 °C, the reaction continue for 40 cycles at 95 °C for 15 s and 60 °C for 1 min. At the end of the reaction, the results were evaluated using the ABI PRISM 7500 software. For each sample, ΔCt value was calculated as Ct of the target gene minus Ct of the endogenous gene. Subsequently, for each sample, ΔΔCt value was calculated as ΔCt of the sample minus ΔCt of the control sample. Relative quantification was obtained as the mathematical function 2 − ΔΔCt. Based on these calculations, the control sample has a value of 1, taken as 100 %.

### Western Blot

Whole-cell lysates (10^6^ cells for each experiment) were prepared by lysing cells in RIPA buffer (50 mM Tris pH  =  7.5, NP-40, 0.1 % SDS, 100 mM NaCl, 50 mM NaF, 1 mM EDTA) supplemented with a set of protease inhibitors: 10 μg of leupeptin per ml, 10 μg of aprotinin per ml, 1 mM sodium benzamidine, and 1 mM phenylmethylsulfonyl fluoride. Proteins (50 μg) were separated on 12 % polyacrylamide, 0.1 % SDS gel. Then, incubation with a monoclonal antibody specific for each PLC isoform (Santa Cruz, CA) followed. Immunoreactive bands were visualized using the enhanced chemiluminescence method. Experiments were independently repeated at least 2 times for each isoform.

For PLC β2 and PLC η2, positive controls were used to test the efficacy of primers in RT-PCR analyses and of antibodies in Western Blot analyses. For PLC β2 human leucocytes were used as positive controls. For PLC η2, nervous tissue was used as positive control.

### Immunofluorescence analysis of subcellular distribution of target molecules

Immunofluorescence localization of all PLC isoforms was performed on coverslips cultured cells with 18 and 24 h U73122 treated cells and untreated control cells. Cells were washed three times with PBS and fixed with 4 % paraformaldehyde (PFA) in phosphate buffer saline (PBS) for 10 min at 4 °C, followed by three washes with PBS. Cells were incubated with primary antibodies diluted in PBS for 1 h at room temperature. Cover-slips were then incubated with the specific secondary antibody Texas Red or fluorescein-conjugated for 1 h at room temperature. Cells were washed twice with 1X PBS 5 min, then counterstained with 4′,6-diamidino-2-phenylindole (DAPI) fluorescent staining. The slides were visualized images were visualized and captured with an Olympus IX50 inverted fluorescence microscope (Olympus, Tokyo, Japan) and processed using Adobe Photoshop 7.0 software.

### Statistical analysis

For in vitro studies, differences were determined either with two-way repeated measures analysis of variance (ANOVA) (http://www.physics.csbsju.edu/stats/anova_NGROUP_NMAX_form.html) with Bonferroni’s multiple comparison test, or by student’s one tailed t test, using Prism 5.0a software (GraphPad Software, San Diego, CA, USA). A p value <0.05 was considered significant.

## Results

### Cells morphology and growth

Untreated cells reached the confluence after 72–96 h, acquiring the expected morphology: cells were oval to spindle-shaped, without branching cell processes. The number of Cells treated with 1 μΜ U-73122 was decreased and intercellular adhesion was subsequently reduced; moreover, a little percentage of round cells were countered (5 %) after 6 h from treatment. In cultures treated with 10 μΜ U-73122, marked morphology changes were observed in MG-63 cells, acquiring round shape.

**Fig. 1:**
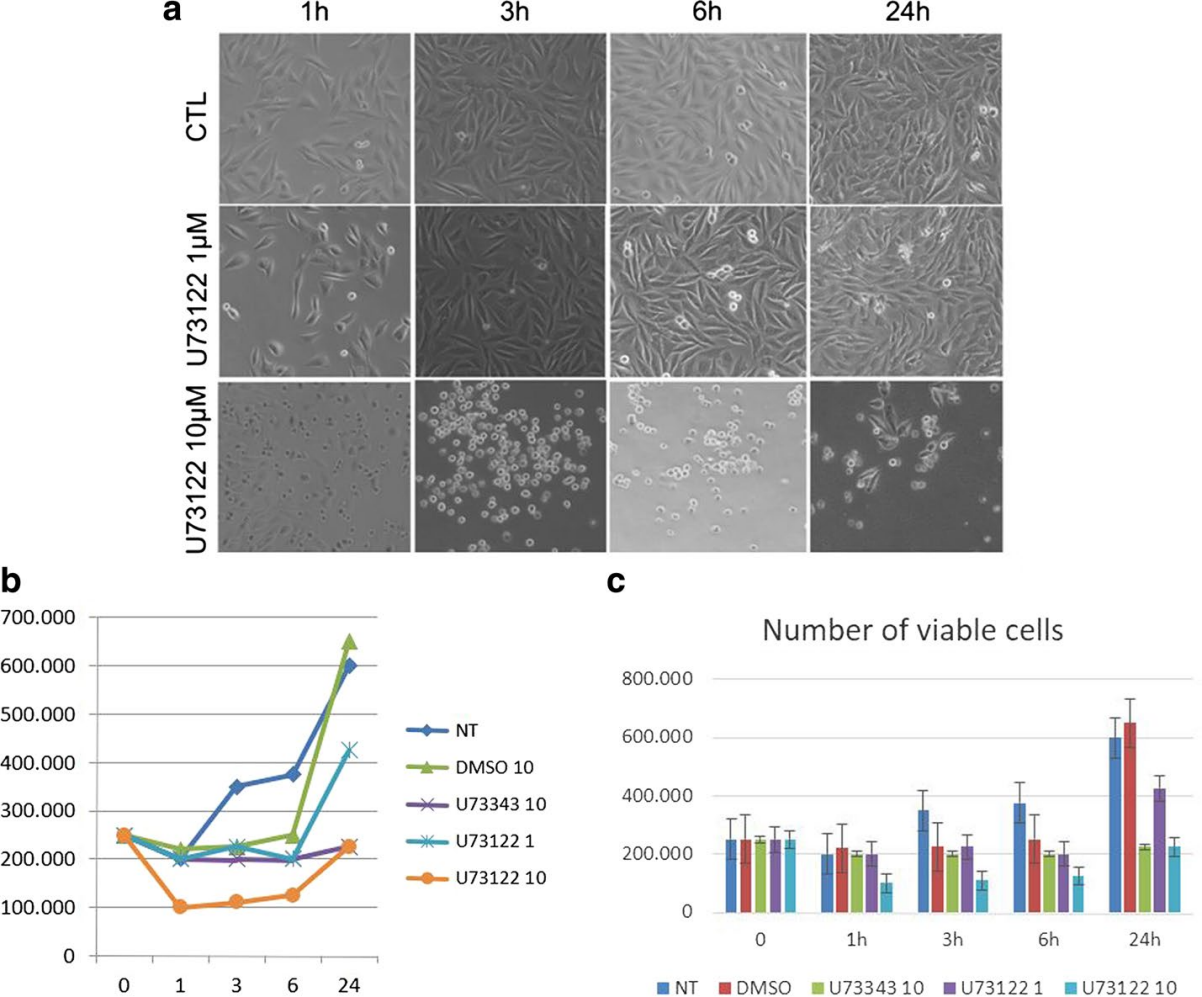
MG-63 cultures. **a** Phase contrast microscopy of MG-63 cells. Cell morphology cultured 24 h: (*upper line*) untreated control cells (CTRL) and cells treated with U-73122 1 μΜ (centre line) and 10 μΜ (*lower line*) after 1, 3, 6 h from seeding **b**, **c** MG-63 cells after 1, 3, 6 h from seeding: untreated (NT), DMSO, U-73122 1 μΜ, U-73122 10 μΜ and U-73343 10 μΜ treated cells. **c** Comparison histogram of cell growth with *error bars*

Untreated cells linearly grew with doubling time about 15 h. After 24 h the number of viable cells increased 2.7 folds (Table 2; Fig. 1).

**Table 2:** Growth data of MG63

MG-63	Time (h)					Mean	SD	SEM
	0	1	3	6	24			
NT	250.000	200.000	350.000	375.000	600.000	423,750	175,849	87,924.57
DMSO	250.000	220.000	225.000	250.000	650.000	225,000	43,301.27	25,000
U73343	250.000	200.000	200.000	200.000	225.000	266,666.67	57,735.03	33,333.33
U73122 1	250.000	200.000	225.000	200.000	425.000	208,333.33	14,433.76	8333.33
U73122 10	250.000	100.000	110.000	125.000	225.000	108,333.33	14,433.76	8333.33

The numbers indicate the viable cells. NT = untreated MG-63. Dosage of exposure to respectively DMSO and DMSO + U73122

DMSO 1 = treatment with DMSO 1 μΜ, DMSO 10 = treatment with DMSO 10 μΜ, U73343 10 = treatment with U73343 10 μΜ; U73122 1 = treatment with U73122 1 μΜ, U73122 10 = treatment with U73122 10 μΜ

*SD* standard deviation, *SEM* standard error mean

In cultures in which DMSO was added to the culture medium, cells showed a little growth slowing down after 1 h from administration of DMSO. However, the number of viable cells rapidly increased after 6 h. The number of viable cells increased 1.9 and 2.8 folds respectively after 1 μΜ and 10 μΜ DMSO. After 24 h from DMSO administration, the doubling time was not completely reached with 1 μΜ DMSO, while it was reached after 13.5 h (Table 2; Fig. 1).

In cultures in which different concentrations of U-73122 in DMSO was added to the culture medium, we observed a significant reduction of the number of viable cells, more evident with the highest U-73122 concentration. The number of viable cells after treatment with 1 μΜ U-73122 was reduced after from 1 to 6 h, and increased 1.7 folds after 24 h (Table 2; Fig. 1).

In cultures in which 10 μΜ U-73343 in DMSO was added to the culture medium, the growth linearly increased from 6 to 24 h. After 24 h, the number of live cells increased 2.5 folds; the doubling time was reached after 16 h (Table 2; Fig. 1).

### Statistical analysis

Mean, standard deviation (SD) and standard error of the mean (SEM) for each cells group are indicated in Table 2.

The unpaired t-test was results are listed in Table 3. Comparing UT versus 10 μΜ U-73122 treated cells resulted statistically significant. Comparing UT versus 1 μΜ U-73122 treated cells resulted not quite statistically significant. Comparing UT versus 10 μΜ U-73343 treated cells, versus 10 μΜ DMSO treated cells and versus 1 μΜ DMSO treated cells resulted not statistically significant (Table 3).

**Table 3:** Statistic evaluation of MG-63 cell viability

	p
UT/10um U73122	0.0292
UT/1um U73122	0.0937
UT/DMSO	0.12
UT/10um U73343	0.2045

p value was considered significant when <0.05

### RT-PCR

Confluence cultured cells: mRNA for *PLCB1*, *PLCB3*, *PLCB4*, *PLCG1*, *PLCD1*, *PLCD3*, *PLCE*, *GAPDH* and *Vil2* was detected. For *PLCG2* the presence of mRNA was variably detected in 50 % of experiments. No mRNA for *PLCB2, PLCD4, PLCH1* and *PLCH2* was detected (Table 4).

**Table 4:** PLC isoforms’ detection in MG-63

	NT								U73122						DMSO			
									30		10							
	T0	1 h	3 h	6 h	24 h	cfl	C−	C+	3 h	24 h	1 h	3 h	6 h	24 h	1 h	3 h	6 h	24 h
*PLCB1*	+	−	−	−	+	+	−	+	−	+	+	−	+	+	+−	+	+	+
*PLCB2*	−	−	−	−	−	−	−	+	−	−	−	−	−	−	−	−	−	−
*PLCB3*	+	+	+	+	+	+	−	+	−	+	+−	+	−	+	+	+	+	+
*PLCB4*	+	+	+	+	+	+	−	+	−	+	+	−+	+	−	+	+	+	+
*PLCG1*	+	+	+	+	+	+	−	+	−	+	+	+	+	+	+	+	+	+
*PLCG2*	+	+	+	+	+	+	−	+	−	+	−	−	−+	−	−	−	+	+
*PLCD1*	+	+	+	+	+	+	−	+	−	+	−+	+	+	−	+	+	+	+
*PLCD3*	+	+	+	+	+	+	−	+	−	+	−	+	+	+	+	+	+	+
*PLCD4*	−	−	−	−	−	−	−	+	−	−	−	−	−	−	−	−	−	−
*PLCE*	+	+	+	+	+	+	−	+	−	+	−+	−	+	−	+	+	+	+
*PLCH1*	−	−	−	−	−	−	−	+	−	−	−	−	−	−	−	−	−	−
*PLCH2*	−	−	+	+	+	−	−	+	−	−	−	+	+	+	+	+	+	+
*GAPDH*	+	+	+	+	+	+	−	+	+	+	+	+	+	+	+	+	+	+
*Vil2*	+	+	+	+	+	+	−	+	+	+	+	+	+	+	+	+	+	+

MG-63 untreated cells (NT) cultured 24 h (T = time; T0; 1, 3, 6 and 24 h). Confl = MG-63 which reached confluence. MG-63 cells treated with U-73122: 30 μΜ (3 and 24 h from treatment), 10 μΜ (1, 3, 6 and 24 h from treatment). MG-63 cultures added with DMSO (1, 3, 6 and 24 h from adding)

Untreated controls: mRNA for *PLCB1* was present at time 0, absent at 1, 3, 6 h and detected after 24 h. mRNA of *PLCB3*, *PLCB4*, *PLCG1*, *PLCG2*, *PLCD1*, *PLCD3*, *PLCE*, *GAPDH* and *Vil2* was detected in all the analyzed times (1.3, 6 and 24 h). mRNA of *PLCB2*, *PLCD4*, *PLCH1* was never detected. mRNA of *PLCH2* was not present at time 0 and after 1 h from cells seeding, while was detected after 3, 6 and 24 h (Table 4).

Control treatment with DMSO 10 μΜ: mRNA of *PLCB1* was variably present after 1 h from treatment and detected after 3, 6 and 24 h. mRNA of *PLCB3*, *PLCB4*, *PLCG1*, *PLCD1*, *PLCD3*, *GAPDH* and *Vil2* was detected after each considered interval. mRNA of *PLCE* was variably detected after 1 h from DMSO addiction in 25 % of experiments, while it was detected after 3, 6 and 24 h. mRNA of *PLCH2* was detected after 1 h, variably present after 3 h in 25 % of experiments and present after 6 and 24 h from DMSO addiction. No mRNA of *PLCB2*, *PLCD4* and *PLCH1* was detected (Table 4).

Treatment with U-73122/DMSO 10 μΜ. *PLCB1*: after 1 h, mRNA was detected, absent after 3 h, present after 6 and 24 h. *PLCB2*, *PLCD4*, *PLCH1*: mRNA was never detected. *PLCB3*: mRNA was detected after 1 h in 50 % experiments, detected after 3 h, undetected after 6 h and detected after 24 h. *PLCB4*: mRNA was detected after 1 and 3 h in 50 % experiments, detected after 6 h and absent after 24 h. The mRNA of *PLCG1*, *GAPDH* and *Vil2* transcription was detected at each interval. *PLCG2*: the mRNA was not detected excepting for detection in 50 % experiments after 6 h from U-73122 adding to the culture. *PLCD1*: mRNA was detected in 50 % experiments after 1 h, and was detected after 3 and 6 h; after 24 h was not detected. *PLCD3*: mRNA was not detected after 1 h, while was present in the remaining intervals. *PLCE*: mRNA was variably detected in 50 % experiments, was absent after 3 h, present after 6 and absent after 24 h. *PLCH2*: mRNA was not detected after 1 h, while was detected in the remaining intervals (Table 4).

### Effectiveness of cells transfection and Real time PCR after ezrin silencing

Silencing of ezrin was validated by western blot, RT-PCR and gel electrophoresis of mRNA extracts and compared to non-targeting control siRNA. Ezrin transcription was compared in cells transfected with ezrin-silencing specific siRNA to controls, comprising untransfected cells and cells transfected with the carrier metefectamine.

Real Time PCR performed after ezrin silencing showed that *Vil2* was not expressed, as expected. *GAPDH* was expressed, as expected. *PLCG2* and *PLCD4* were not expressed, while *PLCE* was quite almost reduced The results are listed in Table 5.

**Table 5:** Real-time results after ezrin (Vil2) gene silencing

		Exp 1	Exp 2	Exp 3
24 h	*Vil2*	Ud	Ud	Ud
48 h	*Vil2*	Ud	Ud	Ud
24 h	*GAPDH*	17,2933769	17,3035622	17,2984695
48 h	*GAPDH*	17,0784683	18,6328697	17,855669
24 h	*PLC E*	26,0919533	26,8768845	26,4844189
48 h	*PLC E*	25,7342033	27,7416077	26,7379055
24 h	*PLC G2*	31,3051376	30,6916809	30,9984093
48 h	*PLC G2*	30,8917274	31,3002968	31,0960121
24 h	*PLC D4*	30,2919121	30,5054932	30,3987026
48 h	*PLC D4*	30,415451	30,8388634	30,6271572

Experiments were repeated each three times (exp 1, exp 2, exp 3). Transfected MG-63 cells (silencing of Vil2)

Ud = undetected until 35 cycles

### Immunofluorescence analysis of sub-cellular distribution of target molecules

**Fig. 2:**
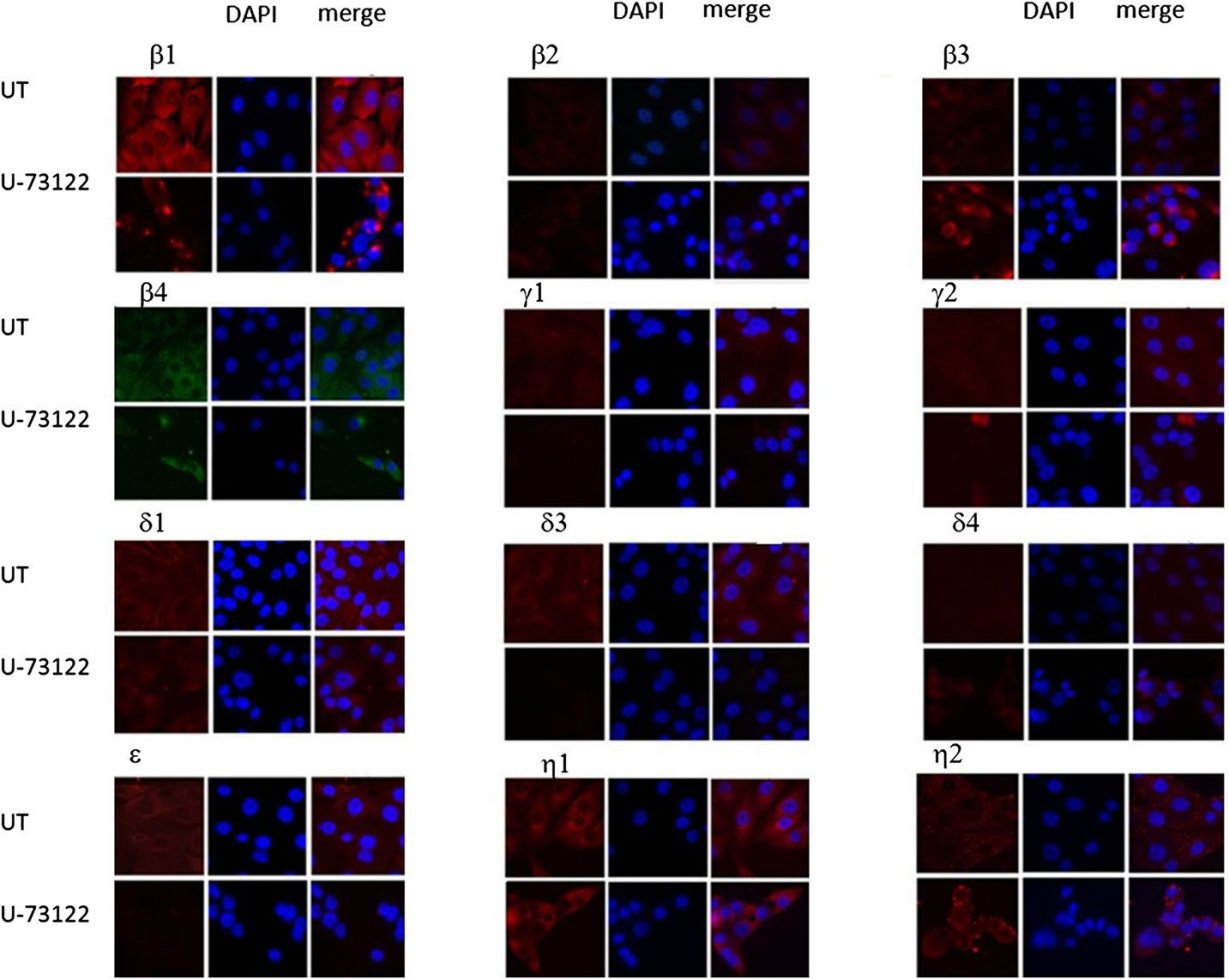
Immunofluorescence images of MG-63 cells (controls and cells treated with 10 micrm U-73122). For each PLC isoform, on the *left* immunomarking with the corresponding antibody Red Texas (*red*) or FITC (*green*) conjugated; DAPI counterstain for nuclei in the centre; on the right merge (inverted fluorescence microscope, 40×)

PLB β1: present in the cytoplasm and weakly in the nucleus of untreated cells, in U-73122-treated cells the fluorescence signal was reduced and the protein was present in perinuclear area (Fig. 2).

PLC β2: a very weak signal was detected in untreated cells; the signal was almost undetectable in U-73122-treated cells (Fig. 2).

PLC β3: in untreated cells, the signal was weakly detected in the cytoplasm; in U-73122-treated cells the protein was distributed exclusively in the perinuclear area (Fig. 2).

PLC β4: in untreated cells, the protein was detected in the cytoplasm, where it was also in U-73122-treated cells, although the intensity seemed to be reduced with respect to controls (Fig. 2).

PLC δ1: in untreated cells, the enzyme was detected in the cytoplasm with evident submembrane reinforcement; in U-73122-treated cells, it was also detected in the cytoplasm, although the membrane reinforcement resulted less evident or absent (Fig. 2).

PLC δ3: in untreated cells, the enzyme was present and localized in the cytoplasm, while in U-73122-treated cells it was not detected (Fig. 2).

PLC δ4: absent both in untreated and U-73122 treated MG-63 cells (Fig. 2).

PLC γ1 and PLC γ2: in untreated cells it was detected in the cytoplasm, where it was detected also in U-73122-treated cells, although the intensity was reduced (Fig. 2).

PLC ε: in untreated cells the enzyme was detected in the cytoplasm, while it was not detected in U-73122-treated cells (Fig. 2).

PLC η1: in untreated cells, the enzyme was localized in the cytoplasm and probably accumulated in plasmatic vesicles; in U-73122-treated cells the intensity of the signal was reduced (Fig. 2).

PLC η2: in untreated cells, the enzyme was localized in the cytoplasm and in plasmatic vesicles; detected in U-73122-treated cells it was localized in the perinuclear area and the plasmatic vesicles resulted less numerous with respect to controls (Fig. 2).

## Discussion

In previous reports, we described the panel of expression of PLC enzymes in MG-63 cells (Lo Vasco et al. 2013). The expressed PLC isoforms were PLC β1, β2, β3, β4, γ1, γ2, δ1, δ3 and ε. In the present experiments we analyzed the sub-cellular distribution of PLC enzymes and the expression panel of PLC isoforms in confluence cultures (72–96 h). In the present experiments, the PLC panel of expression of cells after reaching the confluence did not differ from the 24 h cultured control MG-63 cells. Interestingly, the expression of *PLCG2* transcript was variably detected after confluence (50 % experiments).

In that previous work (Lo Vasco et al. 2013) we had used high concentrations of U-73122, a widely used PLC inhibitor (30 μΜ), observing lack of the PLCs’ transcription after 3 h. The transcripts for the expressed isoforms were observed after 24 h (Lo Vasco et al. 2013). In the present experiments, we confirmed previous observations using 30 μΜ U-73122 (Table 5). U-73122 is not water soluble and, as well as U-73343, needs to be dissolved in DMSO, an amphipathic molecule commonly used as a solvent and worldwide used as cryoprotectant. DMSO acts as a differentiating agent, playing multiple roles both on cellular functions (e.g., metabolism and enzymatic activity) and cell growth by affecting cell cycle and apoptosis (Santos et al. 2003). More specifically, DMSO was demonstrated to induce differentiation in stem and endothelial cells (Jasmin et al. 2010). We aimed to investigate whether DMSO might affect cell growth and/or PLC expression. Thus, we compared the effects of DMSO, U-73122 and U-73343, an inactive U-73122 analogue, upon cell growth, PLC expression and localization. MG-63 cell growth curve was significantly modified by U-73122 (Table 2; Fig. 1). After 24 h from seeding, the number of untreated cells increased 2.7 folds, with doubling time about 15 h. In cultures in which different concentrations of U-73122/DMSO were added to the culture medium, we observed reduction of the number of viable cells. The decrease of viable cells number was statistically significant using 10 μΜ U-73122 dosage. The differences induced in cell growth by the lowest 1 μΜ U-73122 dosage resulted quite not statistically significant. The differences in cell growth after use of DMSO and U-73343 did not result statistically significant compared to untreated cells (Tables 3, 4). Therefore, U-73122 acts upon the cell viability, significantly reducing the number of viable MG-63 cells within 24 h.

U-73122, amphiphilic alkylating aminosteroid homologue of the thiol reagent N-ethylmaleimide (Bleasdale et al. 1990), is the most known and archetypal inhibitor of PLC. U-73122 was described to inhibit the Ca2+ mobilization in a dose-dependent manner, consistent with a mechanism of action involving PLC inhibition. The inhibition of PLC after U-73122 treatment is supposed to be possibly due to an action at Gprotein coupling level (Smallridge et al. 1992). U-73122 was frequently used to define the role of PLC mediated elevation of intracellular calcium concentration, indirectly used as a tool to investigate the PLCs’ signal transduction. However, lack of selectivity of PLC inhibition by U-73122 was described, as it acts (Cenni and Picard 1999) upon a number of unrelated proteins (Feisst et al. 2005; Hughes et al. 2000; Walker et al. 1998; Berven and Barritt 1995; Pulcinelli et al. 1998). U-73122 PLC inhibiting activity is due to prevention of the turnover of PI, thus avoiding the formation of the second messengers IP3 and DAG (Vickers and Fisher 2004; Thomas et al. 2005). However, it is not fully known the mechanism leading to inhibition. Controversial reports described that U-73122 might be ineffective upon inhibition of PLC γ2 (Hellberg et al. 1996). The isomer of U-73122, namely U73343, is commonly used as a control. Although controversial reports might suggest a more complex relationship, U-73343 is considered inactive upon PLC enzymes (Heemskerk et al. 1997). The inhibitory effect of U-73122 appeared to be dependent on the presence of a pyrroledione group, as replacement of this with pyrrolidinedione (to form U-73343) abolished the inhibitory effect. (Hollywood et al. 2010). Both compounds play varying effects on cell signaling by activating nuclear estrogen receptors (Cenni and Picard 1999), acting as a protonophore in rabbit parietal cells, and activating ion channels (Mogami et al. 1997).

In the present experiments, MG-63 cell growth was reduced after U-73343 treatment, although results were not statistically significant (Tables 2, 3, 4; Fig. 1). The present results indicated that U-73122 reduced the cell growth of cultured MG-63 line, decreasing the number of cells from adding to 24 h. Comparisong to other treatments we performed, one might speculate that the cell growth is related to PLC enzymes and that viable cells number is reduced inhibiting PLC enzymes.

Beside the observed effects upon cell viability, previous evidences indicated that U-73122 might act upon the gene expression of PLC isoforms (Lo Vasco et al. 2010a, b, 2011, 2012, 2013). In the present experiments, molecular biology analyses indicated that a rearrangement of the panel of expression of PLC enzymes occurred after 1, 3, 6 and 24 h after U-73122 adding.

In cultured MG-63, *PLCB1* transcript was absent after 1, 3 and 6 h and was detected after 24 h from seeding. In U-73122 treated cells, *PLCB1* transcript was detected after 1 h, absent after 3 and detected after 6 and 24 h. In MG-63 cells grown adding DMSO to the culture medium, *PLCB1* transcript was weakly detected after 1 h from seeding and detected in the remaining intervals. PLC β1 was described to be selectively increased during myoblast and adipocyte differentiation (Faenza et al. 2004; O’Carroll et al. 2009), and evidences suggested that deletion of *PLCB1* favours cancer progression in the myeloid lineage (Lo Vasco et al. 2004; Kaminskas et al. 2005). Thus, increase in PLC β1 protein and *PLCB1* transcript levels might be actually considered to unfavour cancer progression and/or favour differentiation. Our previous results in osteosarcoma cells corroborated the hypothesis that increase of *PLCB1* expression might be opposite to cancer progression, confirming a differentiating role (Lo Vasco et al. 2013, 2014a, b, 2015). However, the increase in *PLCB1* transcription we observed in the present experiments might be due to DMSO, and the role of U-73122 cannot be separated and understood. Moreover, while PLC β1 was localized in the nucleus and in the cytoplasm, after either treatment the protein was reduced and localized only in the peri-nuclear area. The differences in PLC β1 localization, due to DMSO, to U-73122 or to either compounds, might be related to differentiation.

In our present experiments, cultured MG-63 cells own *PLCB3* transcripts and weak *PLC* β3 protein signal was localized in the cytoplasm, accordingly to our previous observations (Lo Vasco et al. 2014a, b). In U-73122 treated cells, no *PLCB3* transcript was detected after 6 h. Interestingly, after U-73122 treatment, the PLC β3 protein was exclusively localized in the nucleus and peri-nuclear area. DMSO did not seem to affect the expression of *PLCB3*. PLC β isozymes are autoinhibited, and several proteins, including Gαq, Gβγ, and Rac1, directly engage distinct regions to release autoinhibition. High concentrations of Gαq or Gβ1γ2 selectively activate PLC β3 at membranes (Charpentier et al. 2014). The transcription factor Stat5, involved in various leukemias, is regulated by PLC β3-dependent manner (Xiao et al. 2010). Moreover, *PLCB3* was claimed to be involved in a subset of endocrine tumours, where its expression was demonstrated to be decreased or absent (Stålberg et al. 2003).

Transcription of *PLCB4* was detected after 1, 3 (in half experiments) and 6 h after U-73122 treatment, and was not detected after 24 h. PLC β4, just as PLC-β1, was specifically involved in the histamine-induced IP3 increases in HeLa cells (Ishida et al. 2014). Amplifications of *PLCB4* were described in glioblastoma multiforme (Waugh 2016) and altered expression was reported in non-small cell lung cancer (Tan and Chen 2014) and endometrial cancer (Orchel et al. 2012). Therefore, PLC β4 might favour the progression of selected cancers. In the present experiments, in MG-63 untreated cells, the protein was detected in the cytoplasm, where it was also in U-73122-treated cells, although the intensity seemed to be reduced with respect to controls.

In our present experiments, *PLCG2* was expressed in 24 h-cultured MG-63 cells, it was not expressed 1 and 3 h after adding DMSO to cultures, and detected in the remaining intervals. After adding U-73122 to MG-63 cultures, *PLCG2* was very weakly detected exclusively after 6 h from treatment, while it was absent in the remaining intervals. The localization of PLC γ2 did not differ, being the protein detected in the cytoplasm, although the signal was dramatically reduced after U-73122 treatment. In our previous studies we had suggested a critical role for PLC γ2 in 143B cells (Lo Vasco et al. 2014a), probably related to their osteolytic features. Literature data indicated that the PLC γ subfamily enzymes used to be detected at higher level in tumour than normal tissues (Arteaga et al. 1991; Noh et al. 1995). Isoforms belonging to the PLC γ subfamily contain a unique region comprising two tandem SH2 domains and one SH3 domain adjacent to a split PH (Katan and Williams 1997), which allow the interaction with different molecules (Bunney and Katan 2011). PLC γ2 is required for early phase osteoclast differentiation (Kertész et al. 2012), and is involved both in actin cytoskeleton reorganization and Rac-activation in dendritic cells (Cremasco et al. 2010), as well as in the integrin-mediated processes of adhesion, migration and bone resorption in osteoclast (Epple et al. 2008). PLC γ2 was also suggested to represent a critical regulator of the cellular and molecular mechanisms occurring in bone and immune cells during autoimmune inflammation (Faccio and Cremasco 2010).

Although U-73122 is widely used as non isoform-specific PLC inhibitor (Lea et al. 2002), it was controversially considered to act upon PLC γ subfamily enzymes (Kim et al. 2012). In fact, isoforms belonging to the PLC γ subfamily are considered “udraggable” proteins (Lattanzio et al. 2013), with special regard to PLC γ1 isoform. However, U-73122 is used as a PLC γ inhibitor (Glassford et al. 2003). Our present results indicate that U-73122 affects the expression of *PLCG* genes.

The results of morphology experiments did not show accountable variations in the presence of the PLC γ2 protein within the U-73122 treated cells, excepting for slight reduction of fluorescence intensity. However, the transcription of PLC γ2 significantly differs after treatment with U-73122 compared with both untreated counterpart and DMSO adding to the cell cultures. Interestingly, ezrin silencing induced loss of *PLCG2* expression according to previous findings in osteosarcoma which suggested a crucial role for this isoform (Lo Vasco et al. 2014a, b).

The transcription product of *PLCD3* was not detected after 1 h from U-73122 treatment, while it was detected in the remaining intervals. PLC δ3, just like PLC δ1 and PLC β1, was localized to the cleavage furrow during cytokinesis. Activation of selected PLC isoforms at the cleavage furrow controls progression of cytokinesis through regulation of PIP2 levels (Naito et al. 2006). PLC δ3 is highly enriched in the cerebellum and cerebral cortex and was demonstrated to promote neurite extension negatively regulating RhoA expression (Kouchi et al. 2011). Moreover it was found altered in nasal polyps (Babeto et al. 2010). PLC δ isozymes, the most primitive and evolutionary conserved, are known to be the most sensitive to calcium (Suh et al. 2008). Our previous studies described the variation of PLC δ1 and PLC δ3 expression in LPS-induced inflammation and inflammatory diseases, such as endometriosis (Lo Vasco et al. 2010a, b, 2011, 2012). That suggested that PLC δ3 might be involved in the fine tuning and regulation of the inflammation cascade. Further studies are required in order to highlight the role of PLC δ3 isoform in the progression of the cell cycle, with special regard to the activity upon proliferation regulation.

In the present experiments, the *PLCE* mRNA was detected in 50 % experiments after 1 h from U-73122 treatment, was absent after 3 h, present after 6 and absent after 24 h, indicating a “fluctuating” behaviour. In untreated MG-63 cells, the enzyme was detected in the cytoplasm, while it was not detected in U-73122 treated cells. PLC ε, expressed in the outermost layer of the neural tube, was widely described in the central nervous system, probably related to neuron differentiation (Lo Vasco et al. 2012). Literature data indicated that PLC ε is important for heart development (Tadano et al. 2005; Wang et al. 2005), is involved in nephrotic syndrome (Hinkes et al. 2006), increases insulin secretion (Dzhura et al. 2011), and is highly expressed in the lung (Smrcka et al. 2012). PLC ε activates and is activated by small G protein Ras/mitogen-activated protein kinase (MAPK) signaling pathway (Lopez et al. 2001). The activity of PLC ε, regulated by association with Ras and Rap (Kelley et al. 2001; Schmidt et al. 2001; Song et al. 2001, 2002), might play a role in intracellular signalling from receptors for fibroblast growth factor (FGF) and various neurotrophic factors involved in the neural development (Vaccarino et al. 1999) and in PC12 pheochromocytoma cells (Qiu and Green 1991; Cowley et al. 1994; Zhu et al. 2002). PLC ε was frequently described to be involved in carcinogenesis. However, controversial observations were reported regarding its role. Evidences support the hypothesis that PLC ε might play a tumour suppressor role in Ras-triggered cancers (Martins et al. 2014). However, silencing of PLCɛ was suggested to induce apoptosis via modulation of bcl-2 and bax in bladder cancer (Zhang et al. 2013) and recent literature data suggest that PLC ε might bear cancer-suppression activity (Martins et al. 2014). Inhibition of PLC ε was suggested to prevent the inflammatory reactions associated with tumour development or inflammatory-related diseases (Wang et al. 2005). *Plce* knockout mice seemed to be resistant to intestinal tumour formation when crossed with Apc∓ mice (Li et al. 2009). Further studies associated selected *PLCE* polymorphisms with oesophageal squamous cell carcinoma (Abnet et al. 2012; Hao et al. 2013) and with gastric adenocarcinoma (Abnet et al. 2012). Knocking down of PLC ε both in vitro and in vivo was referred to inhibit the growth of bladder tumour cells (Cheng et al. 2011; Ou et al. 2010). Overexpression of *PLCE* gene was also reported in murine skin cancer (Bai et al. 2004) and *Plce* (−/−) mice exhibit marked resistance to tumour formation in two-stage skin chemical carcinogenesis (Oka et al. 2010). PLC ε was also claimed to promote progression in head and neck cancer (Bourguignon et al. 2006). Therefore, the mechanism of action and role of PLC ε in cancer promotion and/or progression is far to be highlighted. As several direct effectors of Ras, PLC ε owns RA domain that binds several Ras GTPases, including oncogenic Kras and Hras (Song et al. 2001). The binding involves different interaction surfaces and requires distinct specific recognition of Ras, Rap, or Rho GTPases by at least four different binding interfaces. Those differences have fundamental biological implications for Ras-effector signalling (Song et al. 2001, 2002; Fukami et al. 2010). Interestingly, suppression of ezrin protein expression by antisense transfection or stable expression of short hairpin RNA is associated with reduced Akt and MAPK activity (Khanna et al. 2001, 2004, Yu et al. 2004). In the present experiments, the transcription of *PLCE* gene was affected by ezrin silencing (Table 5), which almost reduced the corresponding transcript, according to previous findings (Lo Vasco et al. 2014a, b). That last observation might confirm the hypothesis that an extensive crosstalk among the PLC enzymes occurs in cells (Lo Vasco et al. 2012).

## Conclusion

The present results confirm our previous observations in human osteosarcoma cell lines suggesting that each cell line owns a specific PLC panel of expression and that a complex organization of PLC enzymes occurs. Probably, PLC enzymes influence each other, networking in a complex manner, probably following a reciprocal hierarchy of control. However, further studies, addressed to identify the crosstalk and the ordered timing of cell line-specific PLC enzymes recruitment might help to highlight the specific role of each isoform, opening the way to novel insights in the progression of the disease, with special regard to metastatic spread.

Our present results indicate that adding of U-73122 to the MG-63 cultures can significantly reduce the number of viable cells, thus slowing the growth within 24 h intervals. Although the specific role played by U-73122 upon PLC enzyme inhibition is still controversial, our results suggest that the compound affects the expression of selected *PLC* genes, opening the way to novel adjuvant therapy perspectives.

Our present results indicate that the use of U-73122 in MG-63 cultured cells significantly reduces the growth rate contemporarily inducing different expression of *PLC* genes. That suggests that PLC expression might be related to the cell growth reduction. Interestingly, the expression of *PLC* genes which codify for PLC isoforms supposed to favour cancer progression was reduced, while the expression of *PLCB1*, which was thought to favour differentiation and/or apoptosis, was increased. Further studies are required in order to elucidate the specific role of PLC δ3 and PLC ε. Thus, our present results accord to previous literature data with respect to other cancer types. However, a number of PLC isoforms are differently expressed and lesser are differently localized within the MG-63 cells, so that no conclusive thesis about their specific role can be formulated. Further studies are required in order to highlight the role of PLCs upon cell viability and the extensive cross-talk among the isoforms that probably contributes to regulate and network the PLC enzymes.
